# The effectiveness of functional task exercise and physical therapy as prevention of functional decline in community dwelling older people with complex health problems

**DOI:** 10.1186/s12877-018-0859-3

**Published:** 2018-07-17

**Authors:** Petra C. Siemonsma, Jeanet W. Blom, Hedwig Hofstetter, Ariëtte T. H. van Hespen, Jacobijn Gussekloo, Yvonne M. Drewes, Nico L. U. van Meeteren

**Affiliations:** 1 TNO Healthy Living, Leiden, Schipholweg 77-89, 2316 ZL Leiden, The Netherlands; 2 Department of Public Health and Primary Care (V0-P), Leiden University Medical Center, Postbox 9600, 2300 RC Leiden, The Netherlands; 3 Department of Gerontology and Geriatrics, Leiden University Medical Center, Leiden, The Netherlands; 4 Topsector Life Sciences and Health (Health~Holland), The Hague, the Netherlands; 5 CAPHRI, Maastricht University, Maastricht, the Netherlands

**Keywords:** Functional training, Exercise, Older people

## Abstract

**Background:**

A physically active lifestyle in older people contributes to the preservation of good health. We assessed the influence of physiotherapy on daily functioning among community dwelling older people (75+) with complex health problems identified with screening, versus usual care. We also compared functional task exercise (FTE), with problems prioritized by older people, trained in the home environment, versus usual preventive physical therapy (PPT).

**Methods:**

Design: FTE and PPT were compared in a randomized controlled trial (RCT). Both interventions were compared with daily functioning in an observational study: control group.

Setting/Participants: Community-dwelling persons aged ≥75 years with daily activity limitations enlisted in 83 general practices (*n* = 155).

Interventions: Both intervention groups (FTE, *n* = 76 and PPT, *n* = 79) received individual, 30 min treatments. The control group (*n* = 228) did not get any experimental intervention offered.

Measurements: Groningen Activities of Daily Living Restriction Scale (GARS).

Statistical analyses**:** Linear Mixed Model analysis, correcting for age, sex, baseline scores and clustering by physiotherapist were used to compare the different groups.

**Results:**

At baseline, 74% percent of the intervention trial group was female vs 79% in the control group. Median ages were 83.9 and 84.7 respectively.

The median baseline GARS-score for the control group was 41.0 (25 and 75 percentile): 35.0; 48.0) and 40.0 (25 and 75 percentile: 32.3; 46.0) for the intervention group (FTE + PPT). The mean change over time was 3.3 (2.5; 4.1) for the control group. Mean difference in change over time between the intervention (FTE + PPT) and the control group was − 2.5 (− 4.3; − 0.6) (*p* = .009).

Between FTE and PPT the difference in change was − 0.4 (95% CI: -2.3; 3.0, *p* = 0.795).

**Conclusion:**

An exercise intervention led by physiotherapists may slow down decline in self-reported daily functioning in older persons with daily activity limitations, identified by pro-active case finding.

**Trial registration:**

Netherlands trial register (NTR2407). Registered 6th of July 2010.

**Electronic supplementary material:**

The online version of this article (10.1186/s12877-018-0859-3) contains supplementary material, which is available to authorized users.

## Background

The consequences of aging include gradually diminished daily functioning [1–4], which may develop into dependency, institutionalization and mortality [5]. In 2013, in the Netherlands, approximately 62% of people aged ≥75 years had limitations in performing activities of daily life and 38% had at least one physical disability [6]. Since our populations are aging, these numbers are expected to rise.

However, there is strong evidence that a physically active lifestyle in older people contributes to the preservation of good health [7], and a higher level of physical activity is associated with reduced incidence of disability, more disability-free years [8] and a longer life [9]. Regular physical training was shown to improve daily physical functioning even in frail older people [10–12].

Functional Task Exercise group program (FTE) is an example of an evidence based program that aims at improving daily activities That are most important to the individual older adult [3, 13, 14]. The FTE group program was shown to be more effective for improvement of daily activities than intensive muscle-strengthening training and no training in older people [14].

An example of how cognitive/perceptual/execution aspects are linked in FTE:

Climbing stairs is an eminent functional activity for most elderly people. In contrast to strengthening exercises for the quadriceps, training stair climbing is a task training. Climbing stairs can be made more intensive by increasing the number of stairs or the speed. This will increase the power and stamina. However carrying one or two bags of groceries up the stairs will force a person to adapt the climbing, for example by countering balance disturbances of being unable to use the handrailing. Carrying a basket of laundry will block the view of the stairs and a person is forced to rely more on other perceptual information that usually. Talking or counting will demand attention and therefore influence the performance. Finally, contextual factors will also force a person to adapt their stairclimbing, for example by having to react to other people descending the stairs or by the type of carpet and steepness of the stairs. The aim of functional training is to increase the difficulty in all areas in order to arrive at a very skilled and flexible performance, in this case stair climbing.

We set out to investigate whether FTE applied to community dwelling older people with complex health problems identified by screening, since the vast majority of people aged 50 years and older want to remain in their current residence, also called ‘aging in place’ [15]. This added value of FTE, in slowing down functional decline, was compared to non-protocolized physical therapy and we studied how these two compare to usual care. In order to help clinicians in deciding whether 1) to pro-actively prescribe physical therapy, and 2) FTE should be preferred over non-protocolized physical therapy.

## Methods

We used two separate study designs: a randomized controlled trial (intervention trial group) and a control group (cohort) embedded in a randomized controlled trial. The addition of the control group was not intended at the start but proved to be necessary to be able to compare the two interventions to usual care.

### Intervention trial group

#### Study design

FTE and PPT were compared in an, assessor blinded, randomized controlled trial (RCT), the ‘intervention trial group’ performed from August 2010 to April 2012.

#### Study population

The target population, aged ≥75 years, was selected from 24 primary care practices in the Western part of the Netherlands. The general practitioners (GPs) excluded persons admitted to a nursing home, with a life expectancy of less than three months, or who did not speak Dutch or were otherwise considered not eligible to participate, eg suffering from serious psychiatric illness. Additional exclusion criteria were inability to comprehend and follow instructions and current physical therapy treatment. The remaining persons were invited by their GP by mail to complete a screening questionnaire. (Additional file 1: Appendix 1) The screening questionnaire consisted of 21 items, covering four domains of health: functional, somatic (health and illness), mental and social. The questionnaire has been shown to predict functional decline [16]. A positive answer to ≥2 questions in a domain led to a positive score on the domain. The ISCOPE (Integrated Systematic Care for Older PEople)- screening questionnaire (control group) had a question about managing finances instead of the capability to climb stairs. Non-responders were reminded by telephone and were offered assistance to complete the questionnaire.

Respondents with a positive score on the functional domain and at least one other domain were invited for a further screening home-visit, performed by research assistants. The aim of the screening visit was to obtain socio-demographic and baseline data and to verify the following eligibility criteria: a positive score on the functional domain and at least one other domain (somatic, mental, social), not receiving physiotherapy treatment, a score of > 18 on the Mini Mental State Examination (MMSE) [17], and to check absolute and relative contra-indications for physical exercise according to the Guidelines for Exercise Test Administration’ in de ACSM Guidelines for Exercise Testing and Prescription [18]. Eligible older people were invited to participate in the randomized intervention study.

#### Randomization

Study participants were randomly assigned to the intervention conditions. A random number sequence was generated using the software environment R version 2.14 [19].

#### Interventions

Both FTE and PPT consisted of individual treatments (30 min) for a maximum of 18 treatments within three months, and were provided to participants with the aim of preventing age-related functional decline. Therapists for either FTE or PPT were not working in the same practice to avoid contamination. In only one out of 28 practices treated both intervention were provided, but by different physiotherapists. FTE was provided in the participant’s home. For more information on FTE: see Additional file 2: Appendix 2. Physiotherapists in the FTE group received extra training for this type of intervention.

The participants in the PPT group were referred to a regular physical therapist. The location of treatment was up to the therapists’ professional opinion. No additional training for the physical therapist for PPT was provided. Twenty percent of therapists already had additional training in elderly care, varying from a course in falls prevention to a master in geriatrics. Therapists received an open referral to help this person with their daily functioning. Any exercises therapy and advise was up to the therapists discretion. Treatment was according to protocols of The Royal Dutch Society for Physical Therapy.

### Observational study

#### Study design

The interventions were compared with the natural course of daily functioning in an observational study design among participants of the ISCOPE-study (trial registration NTR1946) [20], the ‘control group’. The ISCOPE study is a cluster randomized trial among persons aged ≥75 years from 59 general practices in and around the city of Leiden, who were invited to participate (inclusion period September 2009 to September 2010).

#### Study population

To compose the control group with patient characteristics comparable to the intervention groups, participants were selected from 59 primary care practices (*n* = 12, 066 eligible older people) who participated in the ISCOPE-study. We selected respondents without missing questionnaires at baseline or at twelve months of follow up, and who did not receive an intervention that was part of the ISCOPE study (*n* = 4133). Participants who had a positive score on the functional domain and at least one other domain (somatic, mental, social), did not receive physical therapy, and had a score of > 18 on the MMSE were selected [21]. Subsequently, frequency matching was used to obtain the same distribution of scores on the number of domains according to the screening questionnaire as in the intervention trial group.

#### Measurements

The intervention trial and the control group used the same outcome measures. Assessments of outcomes were taken at baseline (T0) and 12 months after baseline (T1) in the participant’s home by independent allied health professionals blinded for group and study (RCT or control group) allocation/origin. For intervention trial participants, information on the total number of treatments and treatment location were collected from the providers of FTE and PPT.

The primary outcome was self-reported functional ability in activities of daily living (ADL). We used the Groningen Activities Restriction Scale (GARS), an 18 item questionnaire that assesses disabilities in competence in ADL, which is validated in the Dutch population [22]. A sum score was calculated ranging from 18 (competent in all ADL activities) to 72 (unable to perform any activity without help), with higher scores indicating more difficulty in performing ADL.

We added the Modified Katz-15 score of independence in ADL [23], a 15 items questionnaire scoring 1 (yes I need help) or 0 (no I don’t need help) for each item. The total sum of the items was used, with a higher sum score indicating more problems in carrying out activities in daily living [23, 24]. This questionnaire is internationally well-known and was added to perform a sensitivity analysis.

#### Sample size calculation

For the intervention trial the sample size calculation was based on the findings of a previous study [16] indicating that at least 64 participants in each group were needed to achieve 80% power to detect a medium effect size (Cohen’s d = 0.5) with a significance level (alpha) of 0.05 using a two-sided two-sample t-test. Assuming a drop-out of 15% (post randomization), 75 (= 64/0.85) older people per group had to start with the intervention.

The size of the control group was based on all available persons in the ISCOPE-study control group that met the eligibility criteria.

#### Statistical analyses

All statistical analyses were performed with SPSS version 23. Descriptive data are presented to characterize the study population and treatment characteristics. We used means with standard deviations (SD) for continuous variables that were normally distributed and medians with 25 and 75 percentiles for continuous variables that were not normally distributed. Proportions were used to describe categorical variables. All analyses were performed according to the intention-to-treat principle (i.e., all baseline data and available follow-up data were included). We computed change over time in outcome variables for both outcome measures. A two-sided α of 0.05 was used as significance level.

In the first analysis of the difference in change on the GARS-score, we compared the two groups in the intervention trial with each other. In the second analysis we compared both intervention groups with the control group. We used Linear Mixed Model (LMM) analysis, correcting for age, sex, baseline scores and clustering by physiotherapist. The model contained a variable for time of measurement (baseline and 12 months) and a variable for ‘intervention’. In the first analysis, the estimate for time of measurement shows the mean change in score for the PPT-group, and the estimate for intervention shows the difference in change in score between the PPT and FTE (extra mean change in FTE). In the second analysis, the estimate for time of measurement shows the mean change in score for the control group, and the estimate for intervention shows the difference in change in score (extra mean change in PPT + FTE) between the control group and the intervention trial group (PPT + FTE). We did the same for the sensitivity analysis of the change in modified Katz-15 score. Missing data were accounted for by the statistical techniques used (LMM) [25].

## Results

### Inclusion of participants

#### Randomized controlled trial

**Fig. 1:**
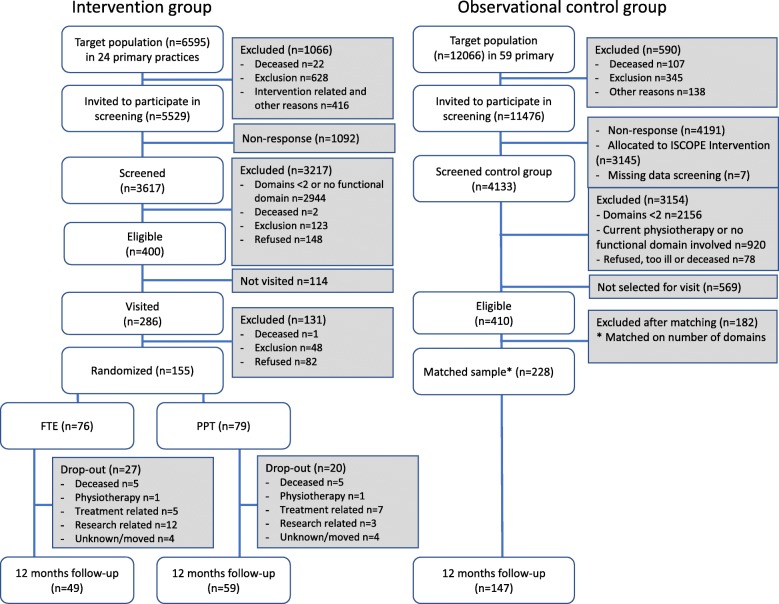
Flowchart

For the FTE and PPT-group 5529 older persons were eligible to participate in the screening. After screening, 400 respondents were eligible to be visited, of which 286 participants were visited, and 114 were not approached and invited within the timeframe of the study due to lack of time. Out of these 286 visited persons 155 agreed to participate in the RCT and signed informed consent, i.e. 54% (155/286) of those invited to participate in the RCT did so (Fig. 1).

Five participants died in each intervention trial group during 12 months, i.e. 6.6% in the FTE and 6.3% in the PPT group. The total drop-out of the trial at 12 months was 35.5% for FTE (*n* = 27) and 25.3% for PPT (*n* = 20) (*p* = 0.167) (Fig. 1).

The average number of participants per physiotherapist was 5 (range 0–12).

#### Control group

In the ISCOPE-study 11,476 persons were eligible to participate in the screening. For the ISCOPE-control group 4133 participated in the screening. Of these, 410 participants were eligible for this analysis. After weighing for distribution of the number of domains according to the screening questionnaire we included 228 control subjects (Fig. 1).

#### Baseline characteristics

Table 1 presents the characteristics of both intervention trial groups and the control group.

**Table 1:** Baseline characteristics in the intervention trial groups (PPT and FTE) and the control group

	Preventive physio therapy	Functional task exercise	Control^a^	*P*-value control vs interventions groups^b^
N	79	76	228	
Sex *(n, % female)*	59 (75)	55 (72)	179 (79)	0.761
Age *(median (25 and 75 percentile)*	83.9 (80.2;86.4)	84.0 (79.4;88.7)	84.7 (80.5;89.5)	0.769
Number of problem domains≥3 (*n (%)*)	58 (73)	53 (70)	164 (72)	0.946
Positive score somatic domain (*n (%)*) Mental domain (*n (%)*) Social domain (*n (%)*)	69 (87) 56 (71) 46 (58)	67 (88) 53 (70) 29 (38)	204 (90) 155 (68) 108 (48)	0.598
MMSE *(median (25 and 75 percentile))*	28 (26; 29)	28 (26; 29)	27 (25; 29)	0.053
GARS *(median (25 and 75 percentile))*	40 (34; 46)	40 (33; 46)	41 (35; 48)	0.308
Modified Katz-15 *(median (25 and 75 percentile))*	5 (3; 7)	5 (3; 6)	5 (3; 7)	0.148

^a^Matched with randomized intervention group for number of problem domains

*GARS* = Groningen Activity Restriction Scale (higher scores indicate more disability), *Modified Katz-15* = Katz Index of independence in ADL (higher sum scores indicate more problems in carrying out activities in daily living)

^b^Chi-square test for dichotomous variables and Kruskall Wallis test for continuous variables

#### Intensity of interventions

The number of treatments was on average 11 in FTE and 12 in PPT treatments per person. The treatment setting for FTE was prescribed to be the participant’s home for all participants, whereas 80% of the PPT clients also received treatment at home.

#### Effectiveness in randomized controlled trial

The mean change over time in the PPT-group for GARS-score was 2.6 points (95% CI: 1.3; 3.8) (*p*-value < 0.001) and the mean change over time in modified Katz-15-score was 0.1 points (95% CI; − 0.2; 0.5) (p-value 0.509), both indicating a 6.5% (baseline median score of 4 (25 and 75 percentile 34; 46)) and 2.0% (baseline median score of 5 (25 and 75 percentile 3; 7)) deterioration in self-reported performance in ADL. No statistically significant different results were observed between the FTE and PPT groups in mean change of either outcome measure after 12 months (Table 2).

**Table 2:** Daily functioning in the intention-to-treat analysis comparing PPT and FTE adjusted for age at screening, sex, baseline daily functioning and clustering by physiotherapist

	Mean change in 1-year follow-up for PPT group (*n* = 79)	*P*-value	Extra mean change in FTE group compared to PPT group (*n* = 76)	*P*-value
GARS total score (95% CI)	2.6 (1.3; 3.8)	< 0.001	−0.4 (− 2.3; 3.0)	0.795
Modified Katz-15-score (95% CI)	0.1 (− 0.2; 0.5)	0.509	−0.4 (− 1.1; 0.4)	0.339

#### Effectiveness in observational study

The control group changed significantly in both GARS and modified Katz-15 scores over time (3.3 (95% CI: 2.5; 4.1) and 0.7 (95% CI: 0.4; 0.9) respectively), indicating that this group deteriorated 8.0–14% during the 12 month follow-up (with a baseline GARS-score of 41 (25 and 75 percentile 35; 48) and a baseline modified Katz-15 score of 5 (25 and 75 percentile 4; 7)). Comparison of the change in scores of the control group to those of the two intervention trial groups showed that the deterioration in the intervention trial groups (FTE + PPT) was significantly less on both GARS and modified Katz-15 scores, i.e. the difference in change of scores was − 2.5 (95% CI -4.3; − 0.6, *p*-value 0.009) and − 0.8 (95% CI -1.4;-0.3, p-value 0.002), respectively (Table 3).

**Table 3:** Daily functioning in the intention-to-treat analysis comparing the control group and PTT+ FTE, adjusted for age at screening, sex and baseline daily functioning

	Mean change in 1-year follow-up for control group (*n* = 228)	*P*-value	Extra mean change in combined PTT + FTE group compared to control group (*n* = 155)	*P*-value
GARS total score (95% CI)	3.3 (2.5; 4.1)	< 0.001	−2.5 (−4.3; − 0.6)	0.009
Modified Katz-15-score (95% CI)	0.7 (0.4; 0.9)	< 0.001	−0.8 (− 1.4; 0.3)	0.002

*GARS* = Groningen Activity Restriction Scale (higher scores indicate more disability), *Modified Katz-15* = Katz Index of independence in ADL (higher sum scores indicate more problems in carrying out activities in daily living)

## Discussion

In this study we found that daily functioning deteriorated slightly, in community-dwelling older persons identified with complex problems, in both FTE and PPT group, but the change over one year did not differ significantly between the two groups. However, this study gives an indication that an exercise intervention led by a physiotherapist (FTE or PPT), might reduce deterioration in daily functioning significantly in comparison to the control group, which received no physiotherapy intervention.

It could be that the treatment ingredients in the PPT-group did not differ as much from the treatment in the FTE-group as had been expected. In the PPT group 80% of treatments were provided in the participants’ home which was unexpected. The number of intervention sessions differed by one session on average per person between the two intervention groups. We don’t expect a negative effect on the outcome from this small difference.

### Comparison to other studies

Daily physical functioning declined in both intervention trial groups and control group during the one year follow-up. This is in accordance with literature, in which there is consensus about the fact that older persons’ health and physical functioning decline with age [3]. An earlier study showed a decline of 3.5 points on the GARS over 1 year in older people of 75 years and older with problems on 3 or more domains on the ISCOPE-questionnaire [20]. Our results suggest a two-third less steep functional decline in both FTE and PPT over one year time span, compared to the control group. This is in agreement with several studies indicating that structured exercise and/or an active lifestyle has a positive impact on older persons with complex health problems [9, 11, 26–30].

The interventions (FTE and PPT) were found to be equally effective, this is in contrast with previous reviews [26, 27] and with the hypothesis we had at the start of the study. However, the findings are in line with two current meta-analyses which state that no definite conclusions on the most effective type of physical therapy can be drawn [28–30].

### Strengths and weaknesses of the study

The systematic recruitment through active case-finding of participants from a large source population representative of community dwelling older people adds to the generalizability of the results. Daily functioning was measured with validated questionnaires by trained allied health professionals during home visits. This increased the reliability and the completeness of the measurements.

A weakness is the fact that only the two intervention arms were randomized for exercise therapy. In spite of matching on complexity of problems there could still be selection bias left due to different eligibility criteria in the intervention groups and the control group: participants in the intervention trial groups (FTE and PPT) could still be more motivated to exercise than participants in the control group because of their active choice to participate. Another weakness is the fact that there was a larger drop out than expected in both intervention groups. In the FTE group more people dropped out because of research related reasons, such as too many questionnaires to fill in. Other reasons were similar. As both groups underwent the same research related procedures we have no explanation for this.

### Implications for clinicians and policy makers

We found that FTE applied to community dwelling older people with complex health problems identified by screening, has no added value compared to non-protocolized physical therapy to slow down functional decline. However, compared to usual care a strategy of exercise therapy for this group of older people might have a beneficial effect on daily function. This study shows that a proactive way of working, i.e. offering an intervention to older people identified by screening, might be beneficial. This offers options for clinicians to be more pro-active in offering exercise therapy to older people. However, the low uptake of the intervention remains a concern, as many older people have to be screened and visited in order to identify relatively few participants for the intervention.

### Future research

So far, it is unclear which components of exercise therapy are effective in maintaining or improving ADL-function of older people. Generally, it seems exercise as such is beneficial. It seems a logical hypothesis that preventive personalized exercise exercise at home is beneficial for functioning in older people. However, the question if therapy is more effective if given at home also still remains open.

## Additional files

Additional file 1: **Appendix 1.** ISCOPE-screening questionnaire. (DOCX 14 kb)

Additional file 2: **Appendix 2.** Description of FTE-therapy. (DOCX 14 kb)

## Abbreviations

ADL: Activities of Daily Living
FTE: Functional Task Exercise
GARS: Groningen Activities of Daily Living Restriction Scale
GP: General Practitioner
ISCOPE: Integrated Systematic Care for Older PEople
LMM: Linear Mixed Model
MMSE: Mini Mental State Examination
PPT: Preventive Physical Therapy
RCT: Randomized Controlled Trial
SPSS: Statistical Package for the Social Sciences
