# Supplementary material for: The effectiveness of functional task exercise and physical therapy as prevention of functional decline in community dwelling older people with complex health problems
Source: BMC Geriatr. 2018 Jul 17;18:164. doi: 10.1186/s12877-018-0859-3 (PMC6050649; doi:10.1186/s12877-018-0859-3)
Supplement: Supplementary file 1 — Appendix 1. ISCOPE-screening questionnaire. (DOCX 14 kb) [file 12877_2018_859_MOESM1_ESM.docx]

**Appendix 1. ISCOPE screening questionnaire**

**Daily life abilities**

These first questions relate to how you function/manage day-to-day life.

You may be helped in these activities by aids such as a stick walking frame or wheelchair.

1. Can you do the shopping without help from anyone else?

*Yes / No*

2. Can you walk outdoors without help from anyone else?

*Yes / No*

3. Can you dress and undress yourself without help from anyone else?

*Yes / No*

4. Can you go to the toilet without help from anyone else?

*Yes / No*

5. Can you climb the stairs by yourself ?

*Yes / No*

6. How well would you say you cope with your general day-to-day life?

*Well / Average /Not at all well*

**Health and illness**

7. Which mark would you give for your physical fitness?

*1 2 3 4 5 6 7 8 9 10*

*Not at all fit Very fit*

8. Do you experience day-to-day problems due to poor eyesight (even if you wear glasses or contact lenses)?

*Yes / No*

9. Do you experience day-to-day problems due to poor hearing (even if you wear a hearing aid)?

*Yes / No*

10. Have you lost weight (more than 6 kg) in the last 6 months unintentionally?

*Yes / No*

11. Are you using more than 4 different kinds of medicine at the moment?

*Yes / No*

12. Have you had a fall in the last month?

*Yes / No*

13. Have you been admitted to the hospital in the last 6 months?

*Yes / No*

**Psychological functioning**

14. Do you feel you have memory complaints?

*Yes /Sometimes/ No*

15. Have you recently felt sad or depressed?

*Yes /Sometimes/ No*

16. Have you recently felt nervous or anxious?

*Yes /Sometimes/ No*

17. Do you feel pretty worthless at the moment?

*Yes /Sometimes/ No*

**Social functioning**

18. Do you feel that your life is empty?

*Yes /Sometimes/ No*

19. Do you feel the lack of a close friend?

*Yes /Sometimes/ No*

20. Do you feel left alone sometimes?

*Yes /Sometimes/ No*

21. Do you feel there are enough people with whom you feel a close connection?

*Yes /Sometimes/ No*
