# Supplementary material for: The effectiveness of functional task exercise and physical therapy as prevention of functional decline in community dwelling older people with complex health problems
Source: BMC Geriatr. 2018 Jul 17;18:164. doi: 10.1186/s12877-018-0859-3 (PMC6050649; doi:10.1186/s12877-018-0859-3)
Supplement: Supplementary file 2 — Appendix 2. Description of FTE-therapy. (DOCX 14 kb) [file 12877_2018_859_MOESM2_ESM.docx]

**Appendix 2: Detailed description of FTE**

The FTE was offered in (and around) the older person’s home on an individual basis, thus enabling therapists to attune to individual problems and the person’s environment. Supplementary functional diagnostics in the domain of daily functioning were done by the therapist. The older person together with the physiotherapist decided which daily activities needed training in order to improve the ability to live independently. These activities were trained using exercises that closely linked cognition, perception and execution of tasks in relation to the older person’s home environment. Training was problem-oriented, situational and task-specific and helped to build confidence. During FTE the physiotherapist monitored progression regularly and adjusted the training intensity accordingly, with the aim of achieving reserves in both physical, perceptual and cognitive capacities. Physiotherapists also engaged the participants’ social environment (partner, volunteer, homecare worker, etc,) in the training. For example by showing what the older person can train themselves and how others can help/stimulate them to undertake (more) daily activities. In addition to the exercises, physiotherapists advised and motivated the older person throughout the training program to be (more) physically active every day both in their home and in their (close) neighborhood. Further description of FTE is available elsewhere (1). The physiotherapists providing FTE were trained and educated (44 study hours) to carry out the intervention.

Reference

1. Fleuren MA, Vrijkotte S, Jans MP et al. The implementation of the functional task exercise programme for elderly people living at home. BMC Musculoskelet Disord 2012;13:128.
